# Neuraminidase Inhibitor of *Garcinia atroviridis* L. Fruits and Leaves Using Partial Purification and Molecular Characterization

**DOI:** 10.3390/molecules27030949

**Published:** 2022-01-30

**Authors:** Muchtaridi Muchtaridi, Rina Fajri Nuwarda, Emmy Hainida Khairul Ikram, Aisyah Saad Abdul Rahim, Amirah Mohd Gazzali, Habibah A. Wahab

**Affiliations:** 1 Functional Nano Powder University Center of Excellence (FiNder U CoE), Universitas Padjadajaran, Jl. Bandung-Sumedang KM 21, Jatinangor 45363, Indonesia; 2 Department of Pharmaceutical Analysis and Medicinal Chemistry, Faculty of Pharmacy, Universitas Padjadjaran, Jl. Bandung-Sumedang KM 21, Jatinangor 45363, Indonesia; rina.nuwarda@unpad.ac.id; 3 Faculty of Health Sciences, Universiti Teknologi MARA, Bandar Puncak Alam 42300, Malaysia; emmy4546@uitm.edu.my; 4 Faculty of Pharmacy, Universiti Teknologi MARA, Bandar Puncak Alam 42300, Malaysia; aisyahsaad@uitm.edu.my; 5 School of Pharmaceutical Sciences, Universiti Sains Malaysia, Minden 11800, Malaysia; amirahmg@usm.my (A.M.G.); habibahw@usm.my (H.A.W.)

**Keywords:** neuraminidase, *Garcinia atroviridis*, influenza virus

## Abstract

Neuraminidase (NA) is an enzyme that prevents virions from aggregating within the host cell and promotes cell-to-cell spread by cleaving glycosidic linkages to sialic acid. The best-known neuraminidase is the viral neuraminidase, which present in the influenza virus. Thus, the development of anti-influenza drugs that inhibit NA has emerged as an important and intriguing approach in the treatment of influenza. *Garcinia atroviridis* L. (GA) dried fruits (GAF) are used commercially as seasoning and in beverages. The main objective of this study was to identify a new potential neuraminidase inhibitor from GA. A bioassay-guided fractionation method was applied to obtain the bioactive compounds leading to the identification of garcinia acid and naringenin. In an enzyme inhibition study, garcinia acid demonstrated the highest activity when compared to naringenin. Garcinia acid had the highest activity, with an IC_50_ of 17.34–17.53 µg/mL or 91.22–92.21 µM against *Clostridium perfringens*-NA, and 56.71–57.85 µg/mL or 298.32–304.31 µM against H1N1-NA. Based on molecular docking results, garcinia acid interacted with the triad arginine residues (Arg118, Arg292, and Arg371) of the viral neuraminidase, implying that this compound has the potential to act as a NA enzyme inhibitor.

## 1. Introduction

Influenza continues to be a significant public health concern since it causes annual epidemics, and has the potential to spark a global pandemic. Globally, annual seasonal influenza epidemics are predicted to result in approximately 3 to 5 million cases of severe illness, and 290,000 to 650,000 respiratory-related deaths [1]. Among the four genera of influenza viruses (influenza types A, B, C, and D), type A is the most pathogenic group of viruses capable of causing severe respiratory illnesses or death [2]. The 2009 swine flu H1N1 and the highly pathogenic avian flu H5N1 are among influenza A viruses that have posed significant health risks in many parts of the world. The World Health Organization determined swine flu (H1N1) to be a pandemic. There were 94,512 confirmed cases in 123 countries, including 112 cases in Malaysia [3]. 

In Indonesia, 200 cases of bird flu (H5N1) have been reported since 2003, with 168 deaths (case fatality rate: 84%) [4]. 

The influenza virus (IV) contains two major transmembrane glycoproteins: hemagglutinin (HA), and neuraminidase (NA), whose antigenic and genetic diversity are used to determine IV subtypes. HA and NA can recognize the same host cell molecule, which is the sialic acid (SA) and play complementary roles during the replication cycle. A well-balanced action of HA and NA is crucial for establishing a productive infection [5]. 

The HA is responsible for the virus’s initial attachment on the surface of cell receptor that is linked with terminal sialic acid. Furthermore, HA aids in the virus’s internalization into the host cell via endocytosis, delivering the nucleoprotein into the cytoplasm. NA functions as a biological scissor, cleaving sialic acid residues from HA and cell surface glycans and facilitating virus movement during entry, and from the surface glycoprotein of newly synthesized virions, allowing the virus to be released from the host and spread to other cells [2].

As well as viruses, NA is also found in bacteria, protozoa, some invertebrates, and mammals [6,7]. Although they differ in binding affinity or substrate preference, they share several conserved domains and structural similarities [6]. NA plays a vital role in influenza virus replication and the presence of the conserved active site residues between species allows effective inhibition by the host immune defense and delay of virus progeny release from the infected cells. This will reduce the virus population and allow time for the host cell’s immunity to eliminate the virus [8]. NA hydrolyzes α-2,3-sialic acid from sugar (galactose) and is also involved in α-2,6-sialic acid-galactosyl hydrolysis, but this is less efficient, especially if the aglycon is weak (sialic acid) [9]. As a result, the inhibition of NA has become the potential target in the design of anti-influenza drugs. 

Oseltamivir (OTV) and zanamivir (ZANA) are two extensively used clinically effective anti-influenza drugs which were developed using the structure-based drug design (SBDD) approach. Both drugs are well-known to be effective in treating influenza in humans, but they do have resistance issues. Consequently, the discovery of new active compounds that are potent against NA but are not susceptible to resistance has become an important goal in the drug discovery of anti-influenza drugs, which is also the goal for this project. 

Most NA inhibitors were developed through synthetic chemistry, as exemplified by oseltamivir [10], zanamivir [11], and peramivir [12]; however, natural products-derived bioactive compounds remain a viable option. The combination of the two approaches may also be possible. For example, shikimic acid, the starting compound in the synthesis of oseltamivir, is not economically feasible to be obtained via chemical synthesis, but can be efficiently isolated from Chinese star anise [13]. Indeed, natural products are have been shown to contribute significantly in the development of potential drug candidates [14]. 

In peninsular Malaysia and Sumatra, Indonesia, *Garcinia atroviridis* (GA) dried fruits (GAF) are used commercially as seasoning and in beverages. The dried fruits, when sliced, are highly acidic. Its biological activities were previously reported, including antitumor, antioxidant, and antimicrobial activity [15]. The root part has also been shown to have anticancer and anti-inflammatory properties. On the east coast of Malaysia, and Sumatra, Indonesia, the leaves of GA (GAL) are eaten as a salad or vegetable. The leaves are wrapped around fish to prevent spoilage in steamed fish. In Sumatra, GAL is used to treat stomach pains caused by pregnancy [16]. A decoction of the leaves and roots of GA is used empirically to treat ear-aches [17]. 

Flavonoids are phytochemicals with a variety of phenolic structures. They are present in many plants, fruits, vegetables, and leaves, with potential applications in medicinal chemistry [18]. Flavonoids possess anti-inflammatory, anticancer, antioxidant [18], and antiviral properties [19,20]. These natural products are well known for their beneficial effects on health, and numerous efforts are being made to isolate the compounds [21]. As reviewed by Badshah et al., some flavonoids exhibit stronger antiviral activity than commercially available drugs in the treatment of viral infections. Flavonoid’s phytochemicals suppress and act on viruses in a variety of ways. They can prevent viruses from attaching to and entering cells, interfering with several stages of viral DNA replication, protein translation, and polyprotein processing. Additionally, they can prevent viruses from being released and continue to infect other cells [20]. The current study aims to investigate the inhibitory activity of isolated compounds from *G. atroviridis* L. against *C. perfringens*-NA and H1N1-NA. A bioassay-guided study was employed to isolate potential NA-inhibitors from GA. Bacterial NA from *Clostridium perfringens* were used as a starting point for bio-guided screening, followed by testing against NA from H1N1 virus.

## 2. Materials and Methods

### 2.1. Materials

#### 2.1.1. Plant Materials

GA dried fruits and leaves were collected in Beruas, Perak. *G. atroviridis* leaves and fruits were oven-dried (40 °C). The specimens were identified in the Department of Biology’s Laboratory of Plant Taxonomy Herbarium, Faculty of Mathematics and Natural Sciences, University of Padjadjaran (Bandung, Indonesia). Maceration with methanol (MeOH, 3 × 3 L, every 24 h) was used to extract dried fruit slices (1.1 kg), and the solvent was evaporated under reduced pressure to yield a concentrated MeOH extract (135.6 g). The MeOH extract (130 g) was partitioned with H_2_O/MeOH (7:3) (300 mL) and extracted with *n*-hexane (1:1), followed by ethyl acetate (EtOAc, 3 × 300 mL), yielding 15.3 g *n*-hexane extract and 20.0 g EtOAc extract. *G. atroviridis* dried leaves (GAL, 220.78 g) were macerated with MeOH (3 × 1 L) to produce green gum (47.4 g). It was partitioned with H_2_O/MeOH (4:1) (250 mL) and extracted with *n*-hexane (1:1), followed by EtOAc (3 × 250 mL). Under reduced pressure, *n*-hexane and EtOAc were removed, yielding a greenish gum weighing 8.5 g and 23.2 g as *n*-hexane extract and EtOAc extract, respectively.

#### 2.1.2. Chemicals Assay

NA from bacteria *C. perfingens*, 2′2-(4-Methylumbelliferyl)-a-D-N-acetylneuraminic acid sodium salt hydrate (MUNANA), and 2-(*N*-morpholino) ethanesulfonic acid (MES) were obtained from Sigma (New York, NY, USA). NA from H1N1 virus was obtained from SinoBio (Shanghai, China).

#### 2.1.3. NA Enzymes Preparation

The bacterial NA stock 100U (*C. perfringens*) (Sigma, N2876, New York, NY, USA) and 600 µL of NA from H1N1 strain A/CALIFORNIA/04/2009 (SinoBio, Shanghai, China) were suspended separately in 1 mL of MES buffer. The NA stock concentration was 200 mU/mL (*C. perfringens*) and 1200 *p*moles/mL (H1N1). The NA solutions were stored at −20 °C unless used immediately. The optimization of NA was performed according to a previously reported study [22]. MUNANA (50 µM), *C. perfringens*-NA (50 mU/mL), and H1N1-NA (45 mU/mL) were determined to be the best concentrations.

### 2.2. Methods

#### 2.2.1. Column Chromatography

Column chromatography was performed using silica gel 60 (Merck, Kenilworth, NJ, USA, 230–400 mesh) and Cosmosil^®^ 75C-18PREP reverse phase columns (150–230 mesh) (Nacalai Tasque Inc., Kyoto, Japan). Thin Layer Chromatography (TLC) was performed using pre-coated silica gel 60 F254 and RP-18 reversed phase C18 (Merck, layer thickness 0.25 mm). The components were detected under UV 254 and 366 nm or sprayed with 1% vanillin in concentrated H_2_SO_4_, followed by heating (40 °C). On a glass plate, preparative layer chromatography (PLC) was carried out using pre-coated silica gel 60 F254 (Merck, 20 × 20 cm, layer thickness 0.25, 0.5, or 1.0 mm).

#### 2.2.2. General Experiments and Spectroscopy

The ^1^H and ^13^C NMR spectra were recorded at 500 MHz using a BRUKER AVANCE III spectrometer (Rheinstetten, Germany), and the values were reported in parts per million (ppm). Depending on the solubility of the compounds, samples (isolated compounds) were dissolved in deuterated organic solvents. Semi-polar compounds were dissolved in deuterated chloroform (CDCl_3_), whereas polar compounds were dissolved in 99% D_2_O. The mass spectra were obtained using an Agilent 1100 Series LC-MSD-Trap-VL spectrometer (Agilent Technologies, Avondale, AZ, USA) using electrospray ionization as the ion source type. FTIR and UV spectra were recorded using IR-Prestige-21 (Shimadzu, Kyoto, Japan) spectrometer, and a UV-Vis spectrophotometer (specord-200, Analytical Jena, Germany), respectively. An electrothermal melting point apparatus was used STUART-SMP10 (Cole Parmer, Staffordshire, UK) to obtain melting points. The rotation index was determined using ADP 120 Bellingham (Bellingham & Stanley, Kent, UK).

#### 2.2.3. Isolation of Compounds from GAF

Since EtOAc extract was the most active against *C. perfringens*-NA and H1N1-NA, it was fractionated to isolate the active compounds. The fractionation of EtOAc extract was performed column chromatography vacuum with gradient system n-Hexane:EtOAc stepwise 10%, (10:0 to 0:10). It yielded five fractions (GF1: 0.9 g, GF2: 0.9 g, GF3: 0.9 g, GF4: 1.6 g, and GF5: 0.9 g). In terms of fractions with activity, only GF3 and GF4 were isolated further to yield GF31 and GF41, respectively. EtOAC was used to purify the GF3 precipitate to obtain GF31 (567.5 mg). GF41 was obtained by precipitating GF4 and washing it with hexane-EtOAc (7:3) to produce a white powder (1.1102 g). The hexane-EtOAc filtrate was evaporated to obtain a solid amorphous yellow GAF1 (254.0 mg). TLC (RP-C18) was used to monitor GF31, GF41, and GF42 using BuOH-CH_3_COOH-H_2_O, 4:1:5 as the mobile phase.

#### 2.2.4. Spectral Data of Garcinia Acid (GM6 or D2)

GF31, GF41, and GF42 all had the same Rf (0.45) and were consequently classified as GAF1. It was obtained in the form of a brownish amorphous solid with a melting point of 176–178 °C (178 °C, [23]). Garcinia acid was characterized as follows: ${\left[\alpha \right]}_{D}^{25}$ = +100° (c = 1, H_2_O); UV(MeOH), λ_max_ at 273 nm. IR-max cm^−1^: 3435 (br, OH), 1801, 1762 (C=O). 1120, 1087 (CO-O). ESI-MS m/z 378.99 [2M-H]^+^ and ESI (pos)-MS m/z 191.1 [M+H]^+^ (calcd for C_6_H_8_O_8_, 190.11). ^1^H NMR (500 MHz, MeOD) δ ppm 2.72 (d, *J* = 17.50 Hz, H-4a), 3.26 (d, *J* = 17.5 Hz, H-4b), 4.31 (s, 3-OH), 4.92 (1H, H-2). ^13^C NMR (500 MHz, MeOD) δ ppm 41.06 (s, C-4) 80.78 (C-3) 86.23 (C-2) 170.16 (C-5) 172.77 (C-1′) 175.86 (C-2′). These results were consistent with the previously published garcinia acid data [24].

#### 2.2.5. Extraction and Isolation of Compounds from GAL

The EtOAc extract (6.3 g) was then subjected to vacuum silica gel column chromatography (1 × 30 cm), and successively eluted with n-hexane-EtOAc stepwise 10%, EtOAc-MeOH (7:3), and MeOH to yield 28 fractions (60 mL each) labelled as GALF1 (1–2), GALF2 (3–18), GALF3 (19–22), and GALFF4 (23–28). These fractions were monitored by TLC silica gel 60 F254 using n-hexane-EtOAc (3:2) as the mobile phase. GALF2 was further subjected to column chromatography with the solvent *n*-hexane-EtOAc (3:2), yielding a green precipitate. This precipitate was washed with *n*-hexane (8.9 mg) to obtain naringenin (GAL1). Acetone was used to dissolve the GALF3 precipitate, which was then crystallized by EtOAc to produce the amorphous GAL2 (22.6 mg). GAL2 had the same Rf as garcinia acid.

#### 2.2.6. Spectral Data of Naringenin (GAL1)

GAL1 was white amorphous, with a melting point of 250–253 °C (250–252 °C, [25]). GAF1 was characterized as follows: ${\left[\alpha \right]}_{D}^{25}$ = −100° (c = 1, MeOH); UV[(MeOH), λ_max_] at 326 and 289. IR-max cm^−1^: 3257, 3404 (br, OH), 1609 (C=O), 2969, 2925 (C=C). ESI-MS m/z: 273 [M+H]^+^ (calcd for C_15_H_12_O_5_, 272.25). ^1^H NMR (500 MHz, DMSO-*d*_6_) δ ppm 2.69 (dd, *J* = 17.10, 3.07 Hz, 1H, H-3eq), 3.27 (dd, *J* = 17.18, 12.77 Hz, 1H, H-3ax), 5.44 (dd, *J* = 12.77, 2.84 Hz, 1H, H-2), 5.89 (s, 1H, H-6), 6.80 (d, *J* = 9, 1H, H-3′, H-5′), 7.31 (d, *J* = 9, 1H, H-2′, H-6′), 8.31 (s, 7-OH), 9.6 (s-5-OH), 12.15 (s, 4′H). ^13^C NMR (500 MHz, DMSO-*d*_6_) δ ppm 41.93 (C-3), 78.39 (C-2), 94.94 (C-8), 95.75 (C-6), 101.73 (C-10), 115.12 (C-3′, C-5′), 128.29 (C-2′, C-6′), 128.82 (C-1′), 157.68 (C-4′), 162.91 (C-9), 163.44 (C-5), 166.62 (C-7), 196.34 (C-4). These findings were consistent with the previously published naringenin data [26,27].

#### 2.2.7. MUNANA Assays

The MUNANA assay was used to evaluate compounds, extracts, and fractions. MUNANA is a fluorescence-based assay that measures the fluorogenic product 4-methylumbelliferone released by the enzymatic activity of influenza virus NA from the substrate 2′-(4-methylumbelliferyl)-D-*N*-acetylneuraminic acid (MUNANA). MUNANA is a reliable method for assessing the inhibitory effects of NA drugs. MUNANA compares uninhibited NA activity of a virus or bacteria to enzymatic activity after incubation with a range of NI drug concentrations, allowing the determination of IC_50_ value as the drug concentration required to reduce NA activity by 50% [28]. Using a micropipette, 25 L of MES buffer was first added to rows A-G and columns 2–12. 50 µL of 1000 µg/mL NA inhibitors were added to the first empty column A-C for one NI and D-F for the second NI. Following that, 25 µL NA was added to each well (row A-G and column 1–12), while 50 µL MES buffer was added to wells in row H (column 1–2) for blank readings. The mixtures were incubated for 30 min in a 37 °C incubator with the plate covered with aluminum foil.

Following incubation, 50 µL of MUNANA substrate was added to each well, whereas 50 µL of NA and MUNANA were added to row H as a positive control (column 3–4). The mixtures were then incubated for an additional hour at 37 °C. After incubation, the reaction was stopped by adding 100 µL of stop solution to each well. Finally, the plate was read using UV excitation on a microplate reader (Turner Biosystem, Sunnyvale, USA).

#### 2.2.8. Data Analysis of Assay

The results were processed using GraphPad Prism v. 5 by fitting experimental data to the logistic graph, which involved percent inhibition over a range of concentrations. The inhibition constant (IC_50_) was determined by plotting the graph at 50% inhibition using nonlinear regression analysis with the GraphPad Prism software (San Diego, CA, USA). On a semi-log plot, the results were plotted as percent inhibition activity versus inhibitor concentration (nM). The inhibitor concentration was expressed as 0.48 to 250 µg/mL, as the concentration of the inhibitor in the final assay volume.

#### 2.2.9. Molecular Docking

The methods of molecular docking simulation were performed from our previous study [29]. The NA protein of subtype N1 in complex with zanamivir (PDB code: 3B7E) was used as the target. This PDB (3B7E) is NA crystal structures with resolution 1.45 Å from protein virus isolated that recombinant NA from the 1918 influenza virus [30]. Molecular docking simulations were performed with AutoDock 4.2 [31].

## 3. Results

### 3.1. Bioassay-Guided Isolation of Active Compounds from GA

#### 3.1.1. Isolation of Compounds from GAF (*G. atroviridis* Fruits)

**Figure 1:**
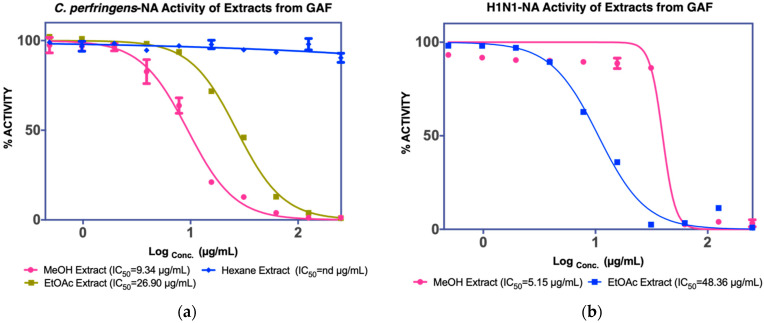
NA inhibition activity of GAF extracts against (**a**) *C. perfringens*-NA, (**b**) H1N1-NA.

The MeOH extract was evaluated against *C. perfringens*-NA, which resulted in an IC_50_ value of 9.43 g/mL as shown in Figure 1. *n*-Hexane extracts were found to be less active against *C. perfringens*-NA, whereas EtOAc extracts were observed to be the most active against both *C. perfringens*-NA and H1N1-NA. Since EtOAc is the most active, it was further fractionated. The fractionation of EtOAc extract resulted in five fractions (GF1: 0.9112 g, GF2: 0.9395 g, GF3: 0.8918 g, GF4: 1.6145 g, and GF5: 0.9011 g).

**Figure 2:**
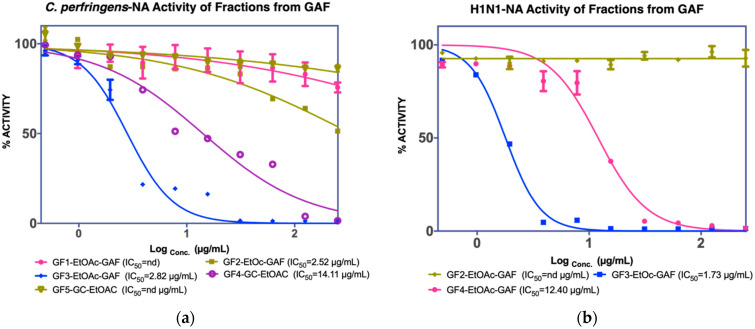
NA inhibition activity of GAF EtOAc fractions against (**a**) *C. perfringens*-NA, (**b**) H1N1-NA.

The assay results revealed that GF2, GF3, and GF4 were active in inhibiting *C. perfringens*-NA. In contrast, as shown in Figure 2, GF2 was ineffective in inhibiting H1N1-NA.

GF3 precipitate was purified using EtOAC to obtain GF31 (567.5 mg). GF41 was obtained by precipitating GF4 and washing it with hexane-EtOAc, yielding a white powder (1.1102 g) and amorphous yellow solid? GF42 (254.0 mg). GF31, GF41, and GF42 showed the same 1D-NMR data (proton and carbon), thus the compounds were labelled GAF1. The crude GAF1 was purified by washing with EtOAc and drying in a desiccator to yield clusters of brownish needle-shaped lactone crystals.

**Figure 3:**
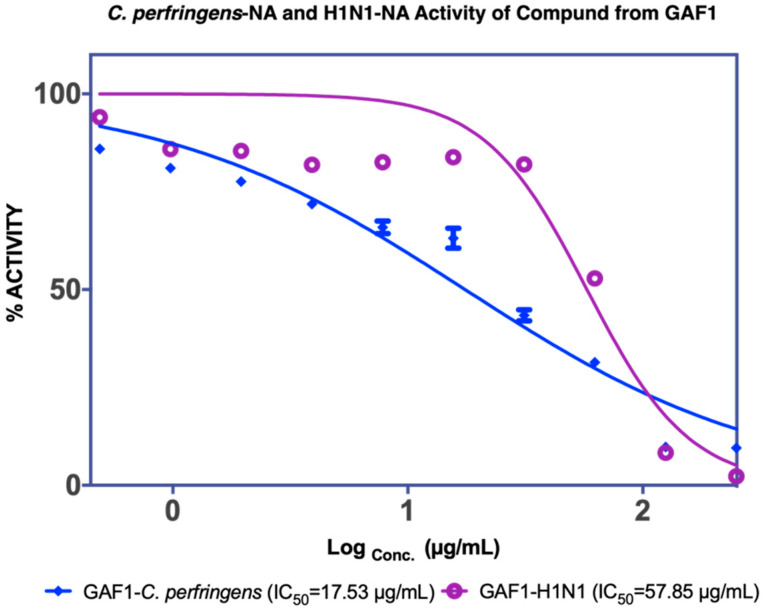
NA inhibition activity of GAF1 isolated compound from EtOAc fractions against *C. perfringens*-NA (blue) and H1N1-NA (purple).

As illustrated in Figure 3, GAF1 was active against *C. perfringens*-NA and H1N1-NA. The IC_50_ values for *C. perfringens*-NA and H1N1-NA were 17.53 μg/mL or 92.21 μM and 57.85 μg/mL or 304.31 μM, respectively.

#### 3.1.2. Isolation of Compounds from GAL (*G. atroviridis* Leaves)

**Figure 4:**
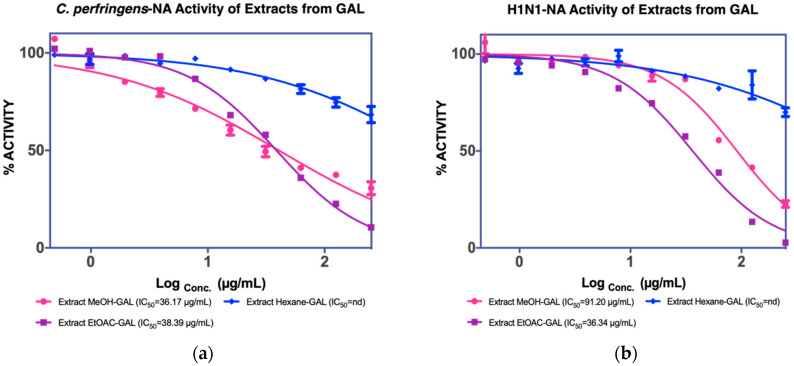
NA inhibition activity of GAL extract against (**a**) *C. perfringens*-NA, (**b**) H1N1-NA.

The MUNANA assay was performed to evaluate both extracts to confirm their activity against *C. perfringens*-NA and H1N1-NA, as shown in Figure 4a. The MeOH extract of GAL demonstrated significant inhibition of *C. perfringens*-NA with an IC_50_ value of 36.17 g/mL and the EtOAC extract exhibited inhibitory activity against both NA (38.39 g/mL), and thus was isolated further to yield four fractions (GALF1, GALF2, GALF3, and GALF4).

**Figure 5:**
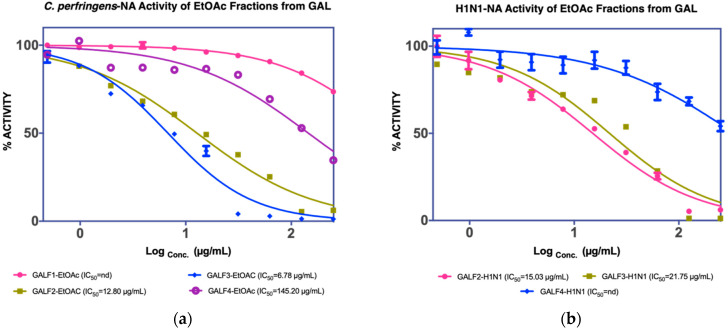
NA inhibition activity of GAL EtOAc fractions against (**a**) *C. perfringens*-NA, (**b**) H1N1-NA.

**Figure 6:**
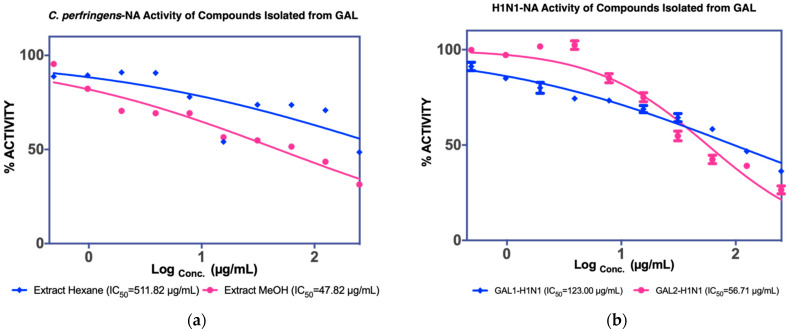
NA inhibition activity of GAL1 and GAL2 isolated compound from EtOAc fractions against (**a**) *C. perfringens*-NA, (**b**) H1N1-NA.

From the four fractions obtained, fractions GALF2 and (1.7772 g) and GALF3 (2.0258 g) showed good NA inhibition on both *C. perfringens* and H1N1, and GALF2 was further isolated to yield GAL1 (8.9 mg) and GAL2 (22.6 mg) (Figure 5). GAL2 has the same Rf as GAF1 (0.45, BuOH-CH_3_COOH-H_2_O 4:1:5) and showed inhibition against H1N1-NA with a maximum inhibitory concentration of 63.7% or an IC_50_ of 56.71 µg/mL, as shown in Figure 6b.

### 3.2. Structure Characterisation

#### 3.2.1. Structure of GAF1 Compound

**Figure 7:**
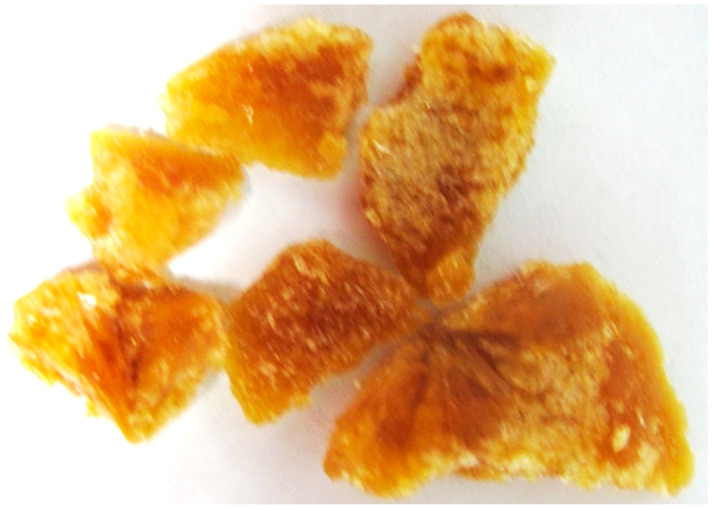
Crystal form of GAF1 compound.

GAF1 crystallized into brownish crystals, as shown in Figure 7.

The IR spectra at wavenumber 3376 cm^−1^ revealed a broad absorption band identified as the carboxylic acid hydroxyl group. Additionally, it was associated with the presence of a carbonyl group at 1734 cm^−1^, while C-O-C ether was detected with a broad signal at 1232 cm^−1^. However, this structure lacked a conjugated system of alkenes or an aromatic group, as evidenced by the absence of a weak stretching vibration at 2800–2900 cm^−1^.

The ^13^C-NMR analysis revealed the presence of six carbons at δ 41.06 (C-4), 80.78 (C-2), 86.23 (C-3), 170.16 (C-1), 172.77 (C-6), and 175.86 (C-1) (C-5). 2D-HSQC findings showed that the chemical shift ^13^C-NMR at δ 41.06 ppm was correlated with the ^1^H-NMR at δ 2.72 and 3.26 ppm, whereas the chemical shift ^13^C-NMR at δ 83.26 ppm was associated with δ 4.31 ppm (3-OH).

**Figure 8:**
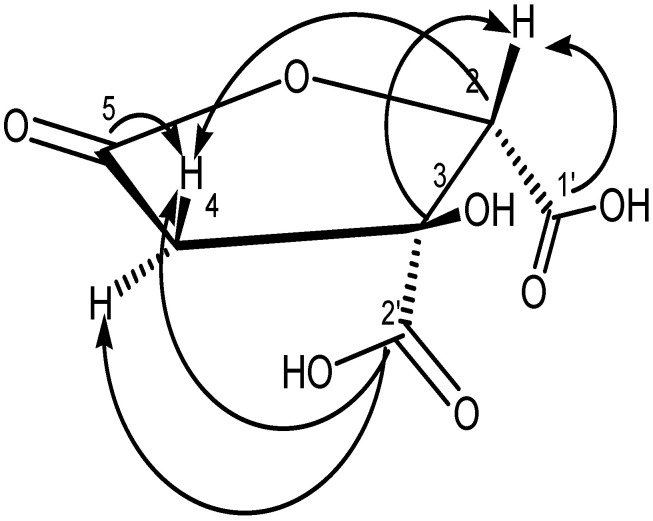
^1^H-^13^C long-range correlations in the 2D HMBC spectrum of GAF1.

The chemical shift at 175.86 ppm in the 2D-HMBC spectrum, as shown in Table S1 and Figure 8, was identified as a carbonyl carbon, which correlated with three-bond correlations with H-4a, H-4b, and H-2 protons. The chemical shift at δ 170.16 ppm was identified as lactone carbonyl of C-5 by correlation (HMBC) between H-2 proton (δ 4.92, s) and H-4a (δ 2.72) and H-4b (δ 3.26). The C-2′ carbonyl of carboxylic acid group was predicted to have a chemical shift at δ 175.86 ppm. The other carbonyl at δ 172.77 ppm was assigned to the other carbonyl of carboxylic acid group (C-1′), which was confirmed by its correlation with H-2 protons (4.92). (s, 1H). The positive-ESI mass spectrum’s molecular ion peak at *m*/*z* 191 [M + H]^+^ indicated that this compound had the molecular formula C_6_H_6_O_7_. Compared to previous studies [24,32,33], GAF1 had the same structure as (2*S*,3*S*)-tetrahydro-3-hydroxy-5-oxofuran-2,3-dicarboxylic acid or garcinia acid, as illustrated in Figure 8.

The NMR data of GAL2 also showed similar spectra as GAF1, and is interpreted as garcinia acid. This was also in accordance with the data reported by Polavarapu et al. [33]. This means garcinia acid can be obtained from the fruits and leaves of *G. atroviridis*.

#### 3.2.2. Structure of GAL1 Compounds

Absorption bands were detected in the IR spectra at 3404 cm^−1^ (-OH); 2967 cm^−1^ (sp2 C-H stretching); 1609 cm^−1^ (>C=O); and 1460 cm^−1^ (C=C stretching aromatic). The UV spectra indicated the presence of phenolic derivatives at 225, 289, and 326 nm [34].

Two signals at δ 2.69 (3-Hα, d, *J* = 17.0) and 3.27 ppm (3-Hβ, dd, *J* = 17.0) were attributed to C-3. The pattern of 1,2,3-tetrasubstituted ring A was generated by proton at 5.89 (H-6, *J* = 2.95) and 5.64 (H-8, *J* = 2.95). The doublet peak signals at 7.31 (*J* = 9.0) and 6.80 (*J* = 9.0) in ring B were assigned to H-2′ or H-6′ and H-3′or H-5′, respectively. The ^1^H-NMR spectrum data were not observed by the ^1^H-NMR signals, despite the presence of the phenolic group indicated by the UV and IR spectrum.

The presence of fifteen carbons was confirmed by the ^13^C NMR spectrum. DEPT90 demonstrated seven methylene carbons at 78.39 (C-2), 94.93 (C-8), 95.75 (C-6), 115.12 (x2) (C-3′ and C-5′), and 128.29 (x2) (C-2′ and C-6′), while DEPT135 revealed one methylene carbon at 41.93. (C-3). Six quaternary carbons were found at 128.82 (C-1′), 157.68 (C-4′), 162.90 (C-9), 101.73 (C-10), 168.48 (C-5), and 170.88 (C-5) (C-7). The presence of carbonyl (C-4) was indicated by the signal at 197.2. There were correlation signals in 2D-HMBC between C-2 at δc 78.39 and δH 7.31 (H-2′) and 3.27 (H-3a) and 2.69 (H-3b); C-4 at δc 196.34 and δH 3.27 (H-3a) and 2.69 (H-3b); C-6 (95.75) and δH 5.64 (H-8); C-9 (162.90) and H-2′ (7.31); C-4′ (157.68) with 7.31 (H-2′) and 6.80 (C-3′); C-5′ (115.12) with 6.80 (C-3′) and 7.31 (C-2′); C-6′ (128.29) with 5.44 (H-2) and 7.31 (C-2′). The molecular ion peak of ESI-MS at m/z 273.2 (M+H^+^) indicated that the structure corresponded to the molecular formula C_15_H_12_O_5_.

**Figure 9:**
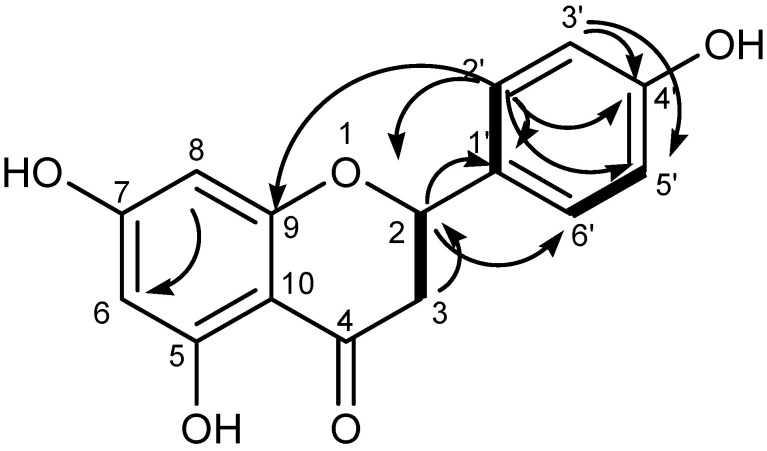
^1^H-^13^C long-range correlations in the 2D HMBC spectrum of compound GAL1.

As shown in Figure 9, the spectroscopic data were consistent with the literature [26,27] for naringenin or 2S-5,7,4′-trihydroxyflavanone, and the NMR data are listed in Table S2.

### 3.3. Binding Interaction of Isolated Compound from G. atroviridis

**Figure 10:**
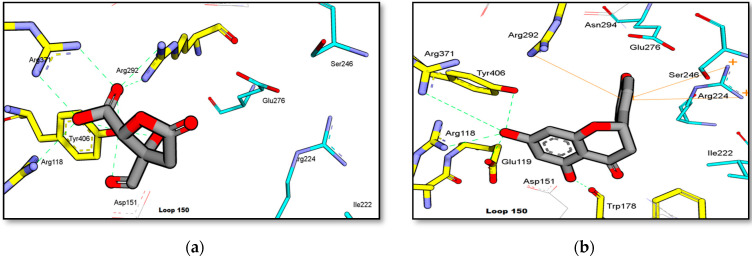
Binding interaction of (**a**) GAF1 (garcinia acid) and (**b**) GAL1 (naringenin) against H1N1-NA (PDB code: 3B7E). (Blue carbon: hydrophobic residue).

The result of bioassay-guided isolation of GA is presented in Table 1. Molecular docking simulation was conducted on GAF1 and GAL1 against NA. As shown in Figure 10, GAF1 and GAL1 interacted well with the active site of NA.

The two carboxylic acid moieties present in the garcinia acid structure may play an important role in its activity against NA. As shown in Figure 10a, the carboxylic acid group at C-2 docked close to the arginine triad with strong hydrogen bonds (2–3 Å) and interacted with Tyr406 at a distance of 2.7–2.8 Å. However, no hydrophobic interaction was observed as it moved away from the hydrophobic residues (Ileu222, Arg224, Ser246, and Glu276).

Naringenin interacted with the arginine triad via hydrogen bonding (with Arg118 and Arg371) and cation-pi interactions (with Arg292), as illustrated in Figure 10b. Arg224 also formed a cation-pi interaction with naringenin’s ring C, but it docked away from the hydrophobic residues Ile222, Glu276, and Ser246, resulting in a less favorable interaction with the NA binding site.

## 4. Discussion

The two compounds isolated from *G. atroviridis* were found to be active against NA. GAF1 and GAL2 have been identified as garcinia acid, while GAL1 has been identified as naringenin. Garcinia acid was found to be more active than naringenin, with IC_50_ values of between 17.34–17.53 µg/mL against *C. perfringens*-NA and 56.71–57.85 µg/mL against H1N1-NA. Garcinia acid and its derivatives have been shown to have biological activities including antifungal [35] and anti-atherosclerosis [36], besides being used as an active ingredient to aid in weight loss [37] or anti-obesity [38] via appetite suppression and inhibition of fat production. They form most of the content in GA’s fruits, and are also known to be present in the root. In addition to garcinia acid, atrovirisidone, naringenin, and 3,8″-bi-naringenin were also previously reported to be isolated from the root, while atroviridin was isolated from the stem bark [39]. Garcinia acid obtained from the fruits (GAF1) and leaves (GAL2) of *G. atroviridis* has previously been isolated from *Garcinia cambogia*, *Hisbiscus cannabinus* [23], *Garcinia indica*, and *Garcinia atroviridis* [40]. Although other pharmacological activities of garcinia acid have been described in the literature, its activity against NA or as anti influenza has never been reported.

Naringenin, on the other hand, is a flavanone derivative with a skeleton similar to xanthone. Citrus and grapefruit are the most common sources of this compound [41]. It was also discovered in the bark of *G. atroviridis*. Naringenin has been shown to have potent antioxidant [42], anti-inflammatory [43,44], antiandrogenic [45], estrogenic [46], monoamine oxidase (MAO)-inhibitor, [41] anticancer-antitumor [47], and anti-dyslipidaemia [44,46] properties. In this study, naringenin was found to have weak activity against NA, with IC_50_ values of 107 µg/mL or 393.38 µM for *C. perfringens*-NA and 123 µg/mL or 452.20 µM for H1N1-NA. This is consistent with Liu et al. (2008) [48] who reported that this compound has a weak NA inhibitory activity with an IC_50_ value greater than 100 µM. However, the authors did not explore the binding interaction in detail. This will be explained further below.

Based on the molecular docking results, the binding free energy of garcinia acid (−8.31 kcal/mol) and naringenin (−8.51 kcal/mol) against NA-H1N1 (3BE7) did not differ much. Both garcinia acid and naringenin interacted with important amino acids via hydrogen bonding, as can be seen in their interaction with the triad arginine residues. These triad arginine residues interacted with the carboxylate of the sialic acid substrate from the virus [49], providing a structural basis in the development of potent inhibitors. However, there was no hydrophobic interactions between garcinia acid and the lipophilic pocket of NA that consisted of several amino acids including Glu276, Ala246, Arg224, and Ile222 [50]. Naringenin has a strong hydrophobic interaction due to the formation of a *pi-pi* cation interaction between its aromatic ring and the hydrophobic residue of NA (orange line) as shown in Figure 10b. This interaction is believed to confer the inhibitory activity of these two compounds against NA [51]. NA inhibition activity of garcinia acid was contributed favourably by the carboxylic acid groups. However, the absence of hydrophobic interaction may be the reason for its weak activity. On the other hand, the lack of carboxylic acid moiety in naringenin can be considered as a significant drawback, thus seem to contribute towards the reduction of the activity reduces the activity of naringenin against NA. This is because of the important interaction that the carboxylic acid moiety contributed in the interaction of a ligand with Arg371, which is considered as the most important residue among the arginine triads that interact with the carboxylic acid moieties of NA inhibitors [52].

## 5. Conclusions

Bioassay-guided fractionation yielded garcinia acid and naringenin from *G. atroviridis* L. fruit and leaves, respectively. Both have inhibitory activity against the NA enzymes. Garcinia acid has a good inhibitory activity against *C. perfringens*-NA, with an IC_50_ of 17.34–17.53 μg/mL, and 56.71–57.85 μg/mL against H1N1-NA. Garcinia acid was found to form a strong ionic interaction with triad arginine based on molecular docking study. The findings in this study provides insight into the ability of *G. atroviridis* to inhibit neuraminidase of influenza virus.

## Figures and Tables

**Table 1:** Highlight of NA inhibition activity isolation of *G. atroviridis* (fruits and leaves).

		Fractions-1	Fractions-2	Compounds
Fruits	MeOH extract IC_50_ *C. perfringens*-NA 9.4 µg/mL IC_50_ NA-H1N1 5.15 µg/mL	Hexane fraction		
		EtOAC fraction IC_50_ *C. perfringens*-NA 26.9 µg/mL IC_50_ NA-H1N1 48.36 µg/mL	F1 Not active	-
			F2 2.82 µg/mL ^a^ nd ^c^	-
			F3 2.82 µg/mL ^a^ 1.73 µg/mL ^b^	Garcinia acid (GAF 1) 17.53 µg/mL ^a^ 57.85 µg/mL ^b^
			F4 2.52 µg/mL ^a^ 12.4 µg/mL ^b^	Garcinia acid (GAF 1)
Leaves	MeOH extract IC_50_ *C. perfringens*-NA 36.17 µg/mL IC_50_ NA-H1N1 36.34 µg/mL	Hexane fraction	-	-
		EtOAC fraction IC_50_ *C. perfringens*-NA 38.39 µg/mL IC_50_ NA-H1N1 48.36 µg/mL	F1 nd ^c^	-
			F2 12.80 µg/mL ^a^ 15.03 µg/mL ^b^	Naringenin (GAL 1) 107 µg/mL ^a^ 123 µg/mL ^b^
			F3 6.78 µg/mL ^a^ 15.03 µg/mL ^b^	Garcinia acid (GAL 2) 17.34 µg/mL ^a^ 56.71 µg/mL ^b^
			F4 145.20 µg/mL ^a^ nd ^c^	

^a^ NA inhibition activity against *C. perfringens*-NA; ^b^ NA inhibition activity against H1N1-NA; ^c^ nd: not detected.

## Data Availability

Not available.

## Supplementary Materials

The following are available online, Figure S1: Spectroscopy data of GAF1, Figure S2: Spectroscopy data of GAL1, Table S1: NMR data of Garcinia Acid and Table S2: NMR data of Naringenin.

## Sample Availability

Samples of the garcinia acid and naringenin compounds are available from the authors.
