# Supplementary material for: Neuraminidase Inhibitor of Garcinia atroviridis L. Fruits and Leaves Using Partial Purification and Molecular Characterization
Source: Molecules. 2022 Jan 30;27(3):949. doi: 10.3390/molecules27030949 (PMC8840166; doi:10.3390/molecules27030949)
Supplement: Supplementary file 1 [file molecules-27-00949-s001.zip › molecules-1492210 SI V4/molecules-1492210 SI V4.pdf]

Supplementary Information

Scheme of Extraction and Isolation Methods

Extraction and Isolation of *Garcinia atroviridis* Fruit (GAF)

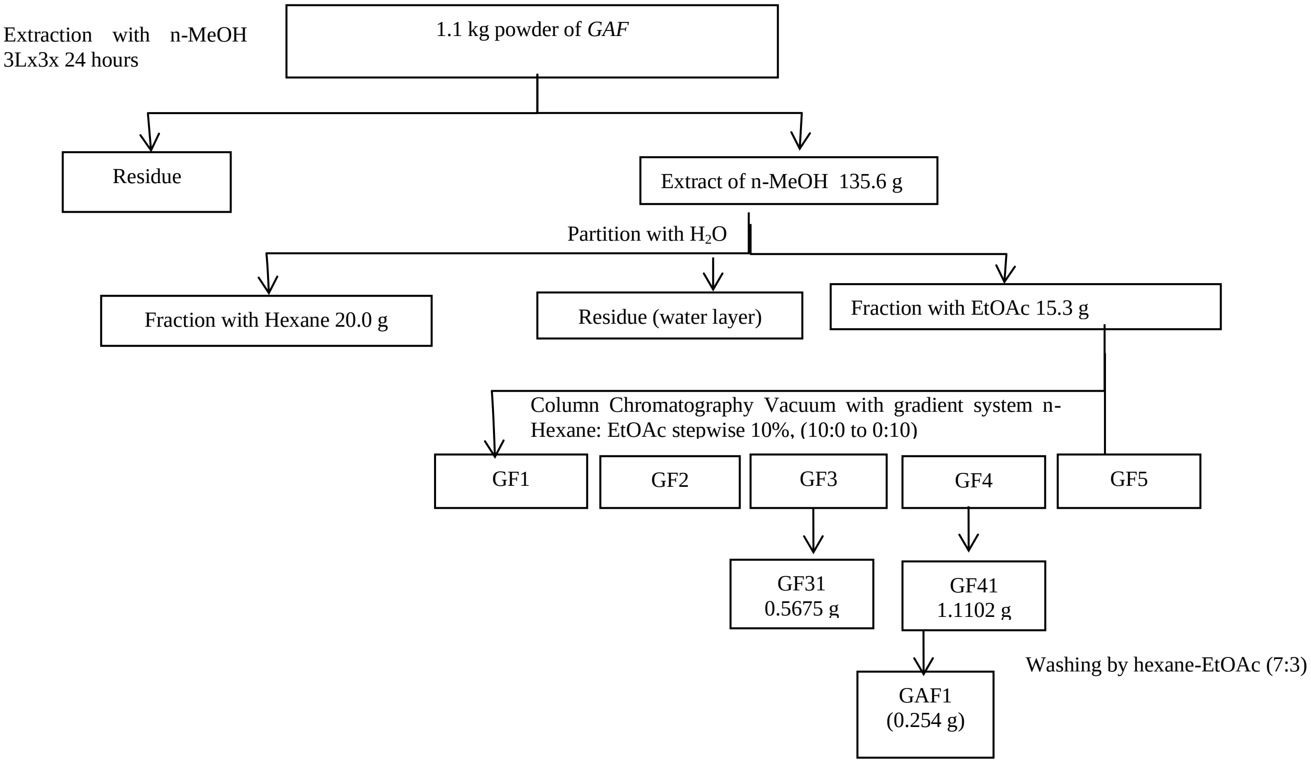

Extraction and Isolation of *Garcinia atroviridis* Leavs (GAL)

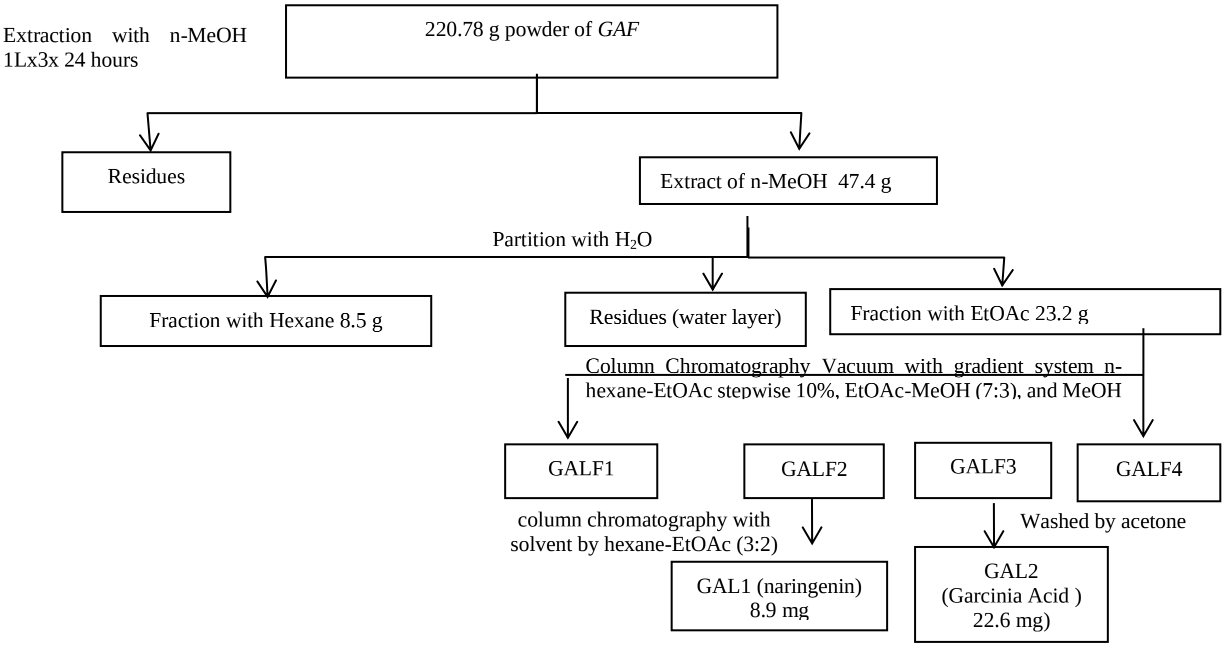

Spectral Data of Garcinia Acid (GM6 or D2)

GAF1was obtained as brownish amorphous solid. Melting point of 176–178 °C (178 °C, [23]). GAF1 was characterised as follows: $[\alpha]_D^{25}$ =+100° (c=1, H_2O); UV(MeOH), λ_{max} at 273 nm. IR-max cm^{-1} : 3435 (br, OH), 1801, 1762 (C=O). 1120, 1087 (CO-O streching). ESI-MS m/z 378.99 [2M-H]+ and ESI (pos)-MS m/z 191.1 [M+H]+ (calcd for $C_6H_8O_8$, 190.11). ^{1}H NMR (500 MHz, MeOD) δ ppm 2.72 (d, *J*=17.50 Hz, H-4a), 3.26 (d, *J*=17.5 Hz, H-4b), 4.31 (s, 3-OH), 4.92 (1H, H-2). ^{13}C NMR (500 MHz, MeOD) δ ppm 41.06 (s, C-4) 80.78 (C-3) 86.23 (C-2) 170.16 (C-5) 172.77 (C-1′) 175.86 (C-2′). These data were consistent with the reported data of garcinia acid (Polavarapu et al., (2011)[33] and Hida et al. (2005))[24].

Table S1. ^{1}H-NMR, ^{13}C-NMR, HSQC and HMBC spectroscopy data (solvent MeOD) of GAF1.

	**1D-NMR**			**2D-NMR**		**Ref** [2]	
**Position**	**^{13}C-NMR**	**^{1}H-NMR**	**DEPT 90**	**HSQC**	**HMBC**	**^{13}C**	**^{1}H**
2	86.23	4.92 (s, 1H)	CH	Yes	H-4a, H-4b	84.5	4.90 (s, 1H)
3	80.78	-			H-2	79.2	
4	41.05	2.72 (d, *J*=17.50 Hz, 1 H, H-4a) 3.26 (d, *J*=17.5 Hz, 1H, H-4b)	-	Yes	H-2	39.4	2.72 (d, *J*=17.50 Hz, 1 H, H-4a) 3.76 (d, *J*=17.5 Hz, 1H, H-4b)
5	170.16		-		H4b	167.0	
1′	172.77		-		H-2	170.5	
2′	175.86		-		H-4b	170.7	
3-OH		4.31 (s, 1H)	-				3.85 (s, 1H)

Spectral Data of Naringenin (GAL1)

GAL1 had white amorphous with mp 250-253°C (250-252 °C, [25]) . GAF1 was characterised as follows: $[\alpha]_D^{25}$ =-100° (c=1, MeOH); UV[(MeOH), λ_{max}] at 326 and 289. IR-max cm^{-1} : 3257, 3404 (br, OH), 1609 (C=O), 2969, 2925 (C=C streching). ESI-MS m/z: 273 $[M+H]^+$ (calcd for $C_{15}H_{12}O_5$, 272.25).. ^{1}H NMR (500 MHz, DMSO-d_6) δ ppm 2.69 (dd, *J*=17.10, 3.07 Hz, 1H, H-3eq), 3.27 (dd, *J*=17.18, 12.77 Hz, 1H, H-3ax), 5.44 (dd, *J*=12.77, 2.84 Hz, 1H, H-2), 5.89 (s, 1H, H-6), 6.80 (d, J=9, 1H, H-3′, H-5′), 7.31 (d, J=9, 1H, H-2′, H-6′), 8.31 (s, 7-OH), 9.6 (s-5-OH), 12.15 (s, 4′H). ^{13}C NMR (500 MHz, DMSO-d_6) ppm 41.93 (C-3), 78.39 (C-2), 94.94 (C-8), 95.75 (C-6), 101.73 (C-10), 115.12 (C-3′, C-5′), 128.29 (C-2′, C-6′), 128.82 (C-1′), 157.68 (C-4′), 162.91 (C-9), 163.44 (C-5), 166.62 (C-7), 196.34 (C-4). These data were consistent with the reported data of naringenin [26,27].

Table S2. ^{1}H-NMR, ^{13}C-NMR, HSQC and HMBC spectroscopy data (solvent DMSO-d_6) of GAL1.

	**1D-NMR**			**2D-NMR**	**Ref (CD_3OD) [6]**	
**Position**	**^{13}C-NMR**	**^{1}H-NMR**	**DEPT135 DEPT90**	**HMBC**	**^{13}C**	**^{1}H**
2	78.39	5.44, (dd, J=12.8, 2.8, 1H)	CH	H-2′, H-3	80.5	5.34 (1H, dd, 13.0 Hz, 3.0 Hz)
3	41.93	2.69, (dd,17.1, 3.1, eq) 3.27 (dd, 17.2, 12.8, ax)	CH_2		44.0	2.70 (1H, dd, 17.0 Hz, 3.0Hz, H-3eq) 3.10 (1H, dd, 17.0 Hz, 13.0 Hz, H-3ax)
4	196.34			H-3, H-3	197.8	
5	163.44				165.5	
6	95.75	5.89 (s, 1H)	CH	H-8	97.1	5.88 (1H, d, 2.0Hz)
7	166.62	-			168.4	-
8	94.94	5.64 (d, J=2.2, 1H)	CH		96.2	5.90 (1H, d, 2.0Hz)
9	162.90	-		H-2′	164.9	-
10	101.73	-			103.4	-
1'	128.82	-		H-2′ H-3′ H-2	131.1	-
2'	128.29	7.31 (d, J=9.0, 1H)	CH		129.0	7.31 (2H)
3'	115.12	6.80 (d, J=9.0, 1H)	CH		116.4	6.82 (2H)
4'	157.68	-		H-2′, H-3′	159.0	-
5'	115.12	6.80 (d, J=9.0, 1H)	CH	H-3′, H-2′	116.4	6.82 (2H)
6'	128.29	7.31 (d, J=9.0, 1H)	CH	H-2, H-2′	129.0	7.31 (2H)
4'-OH		12.15 (s, OH)			-	NO
5-OH		9.6 (s, OH)			-	NO
7-OH		8.31 (s, OH)			-	NO

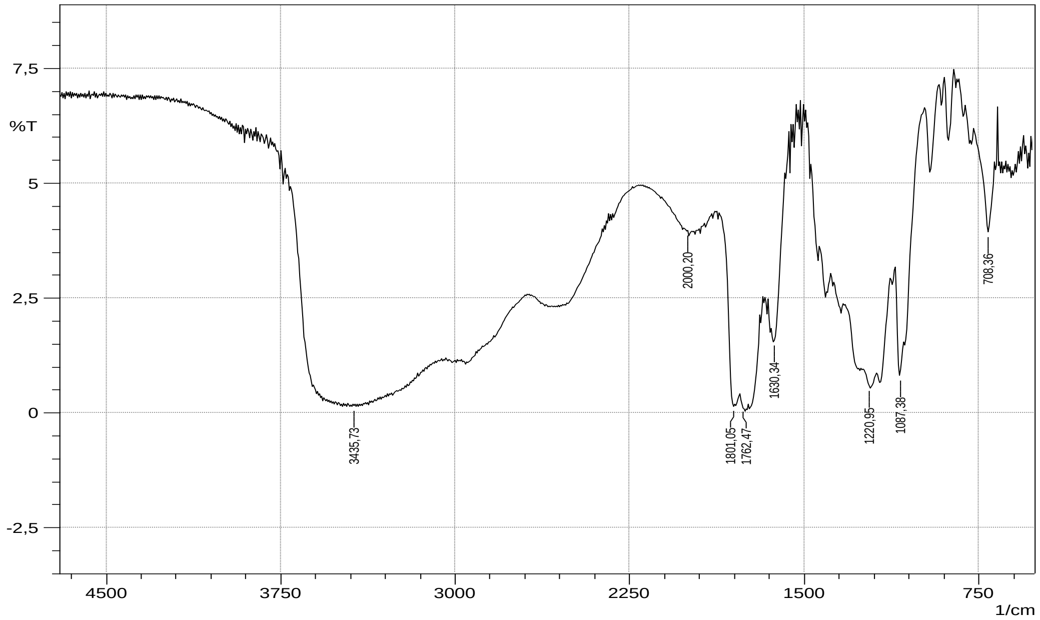

IR spectrum of GAF1 compound that isolated from *G. atroviridis* fruits

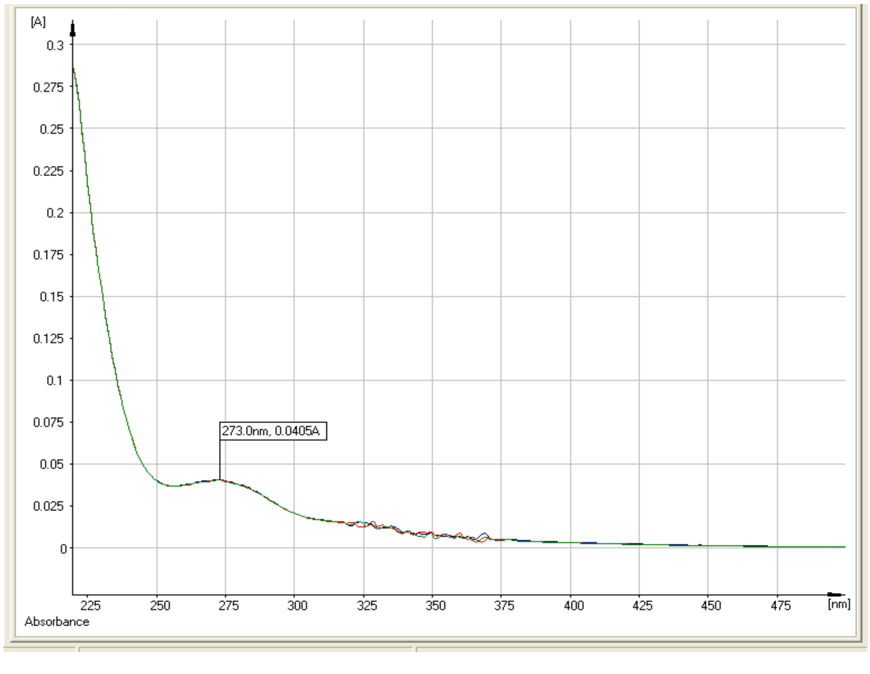

UV spectrum of GAF1 compound that isolated from *G. atroviridis* fruits

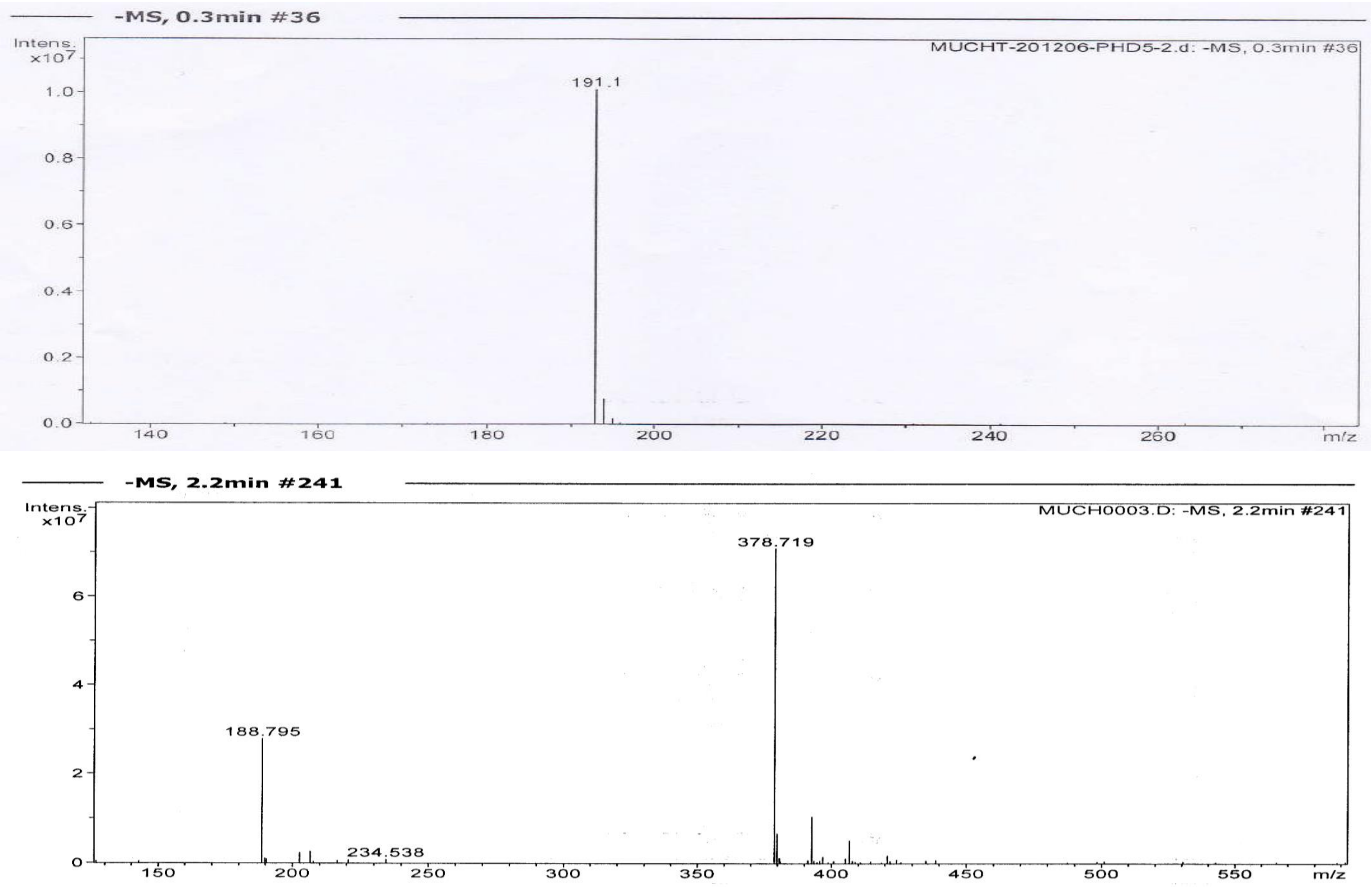

Positive Mode $[M+H]^+$ and Negative Mode $[2M-H]^+$ of Mass Spectrum Using ESIMS-Trap-Direct Injection in MeOH of GAF1 compound that isolated from *G. atroviridis* fruits

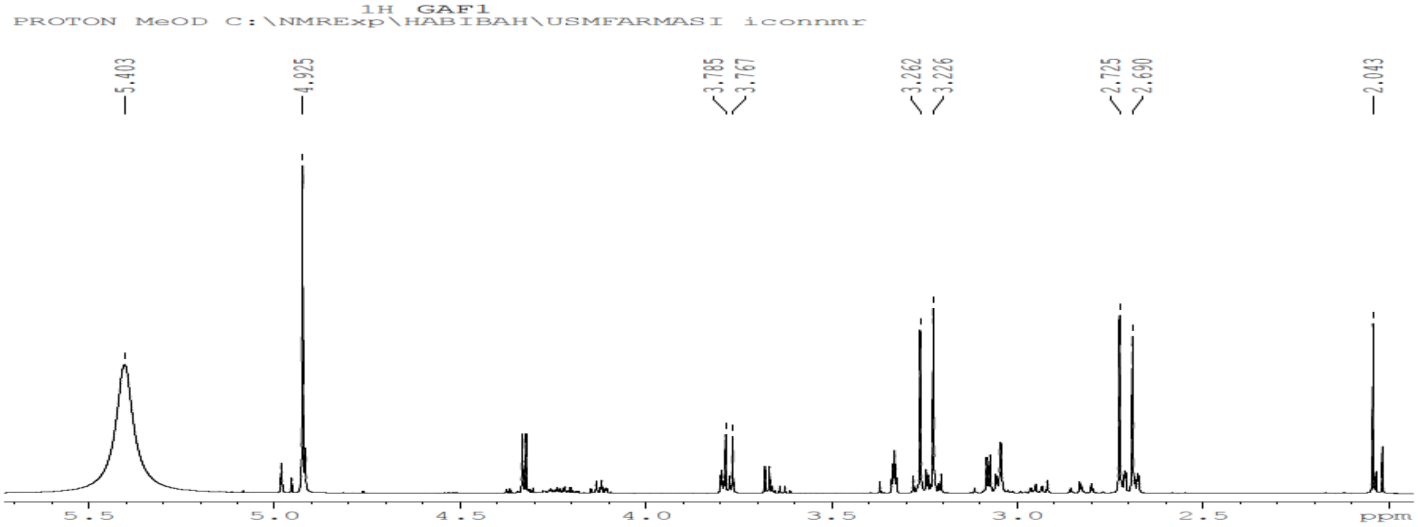

^{1}H-NMR spectrum of GAF1 compound that isolated from *G. atroviridis* fruits using CD_3OD solvent

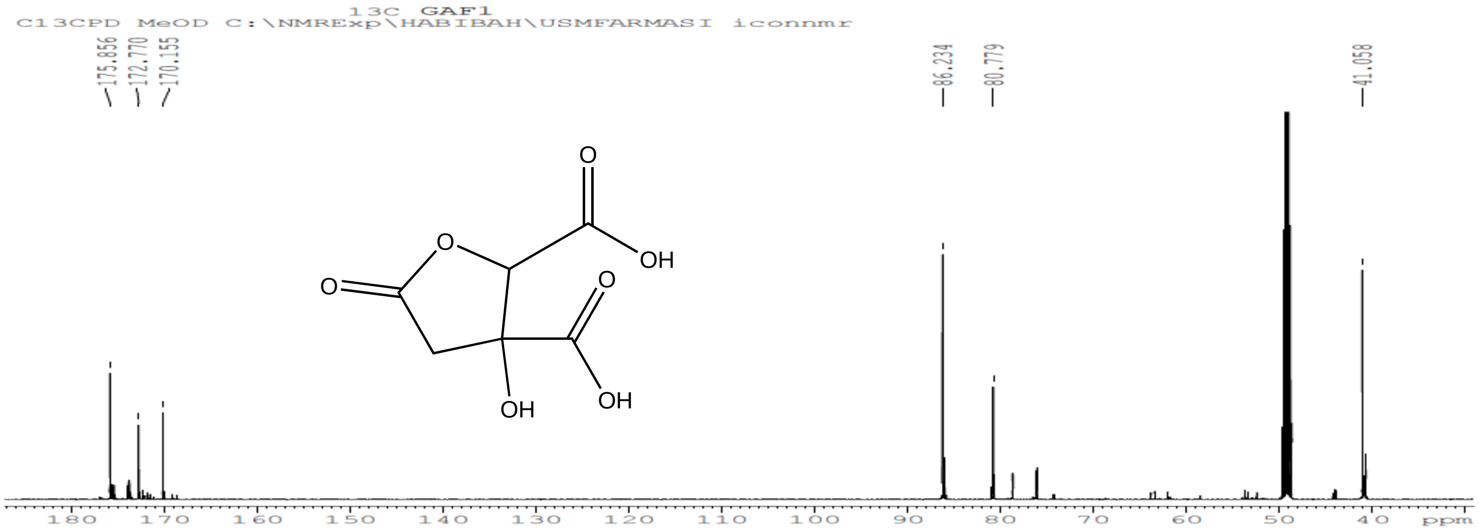

^{13}C spectrum of GAF1 compound that isolated from *G. atroviridis* fruits using CD_3OD solvent

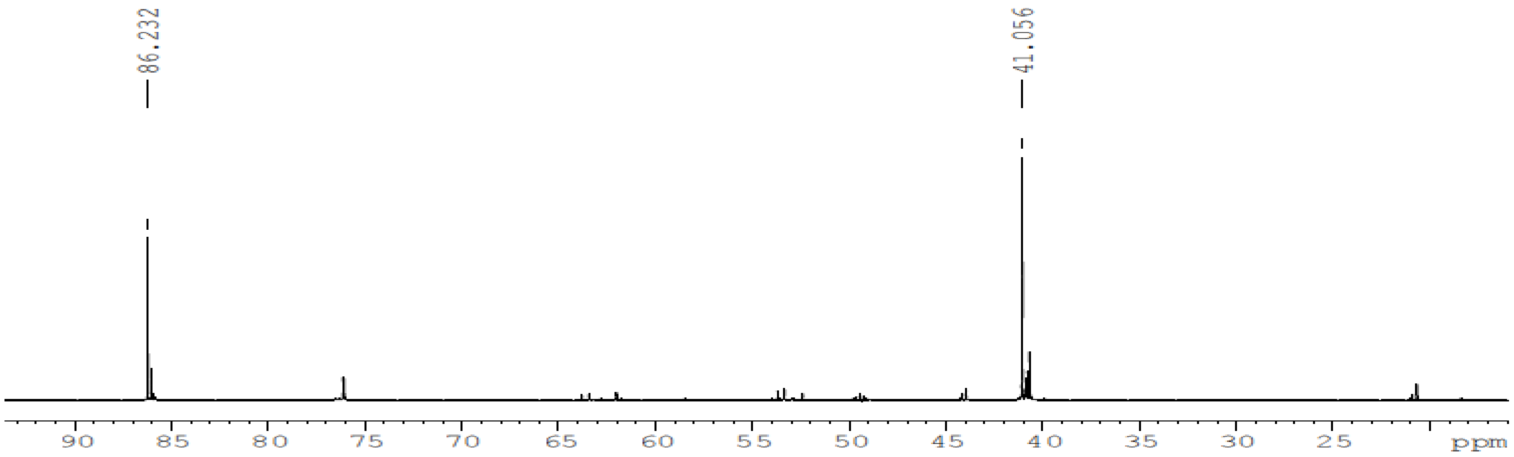

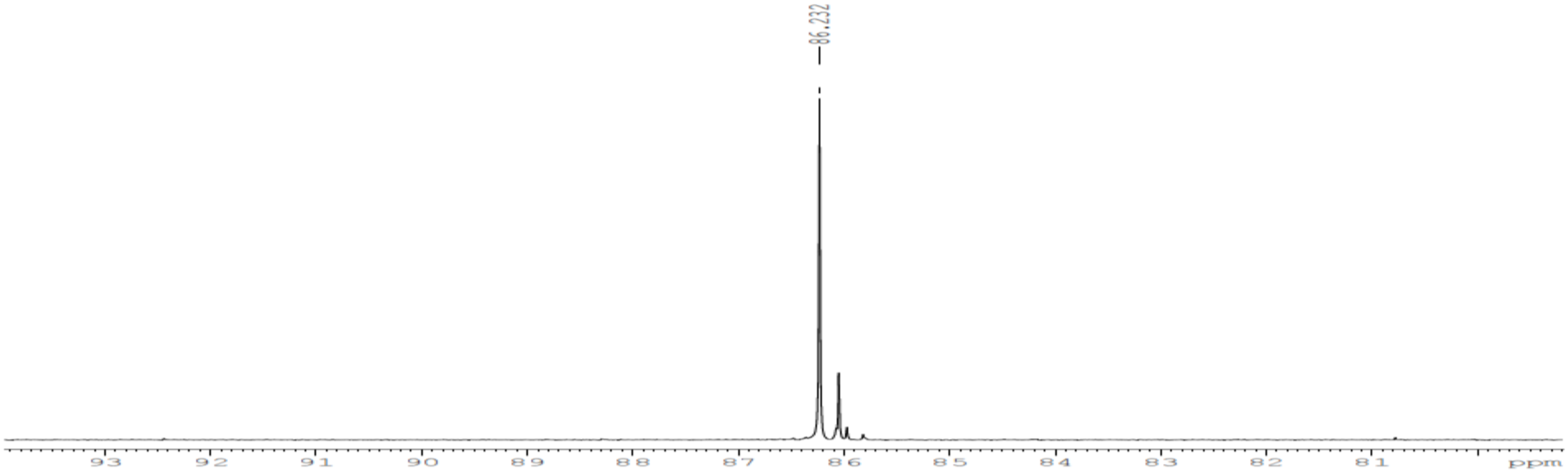

DEPT45 and DEPT90-NMR spectrum of GAF1 compound that isolated from *G. atroviridis* fruits using CD_3OD solvent

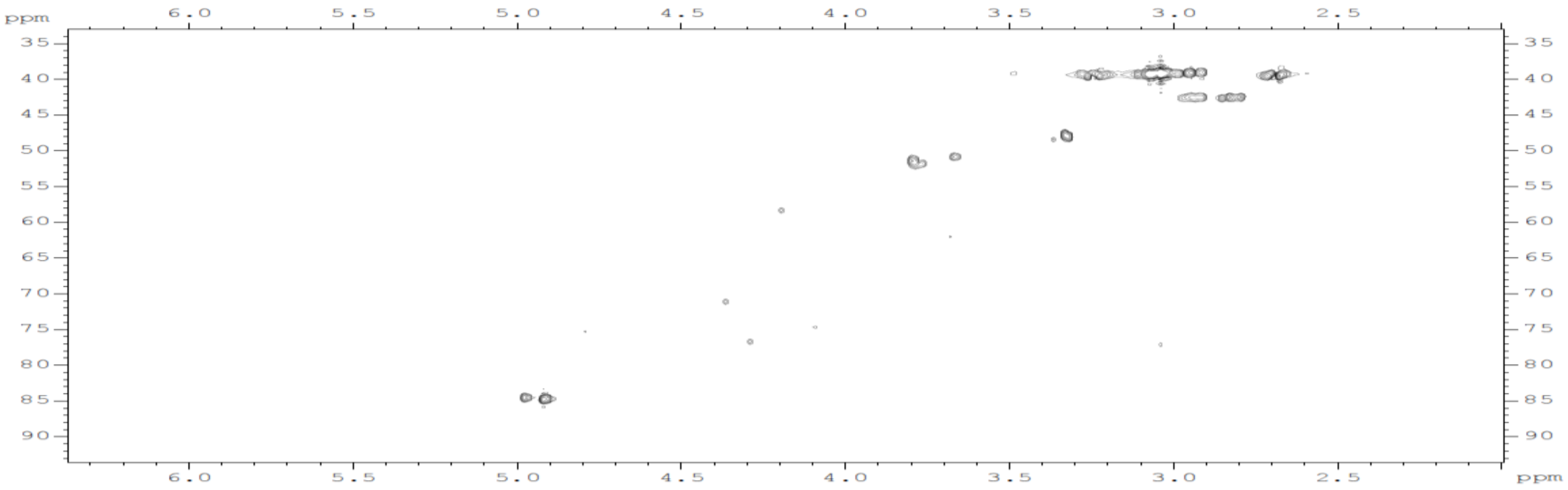

2D-HSQC-NMR of GAF1 using CD_3OD solvent

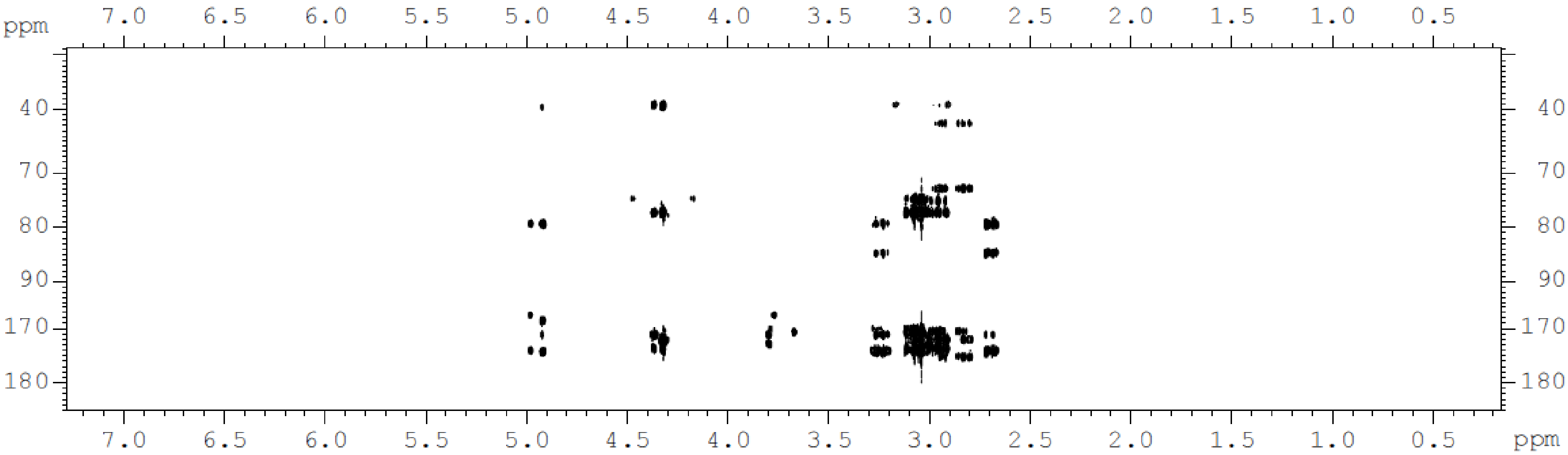

2D-HMBC-NMR of **GAF1** using CD_3OD solvent

Figure S1: Spectroscopy data of GAF1

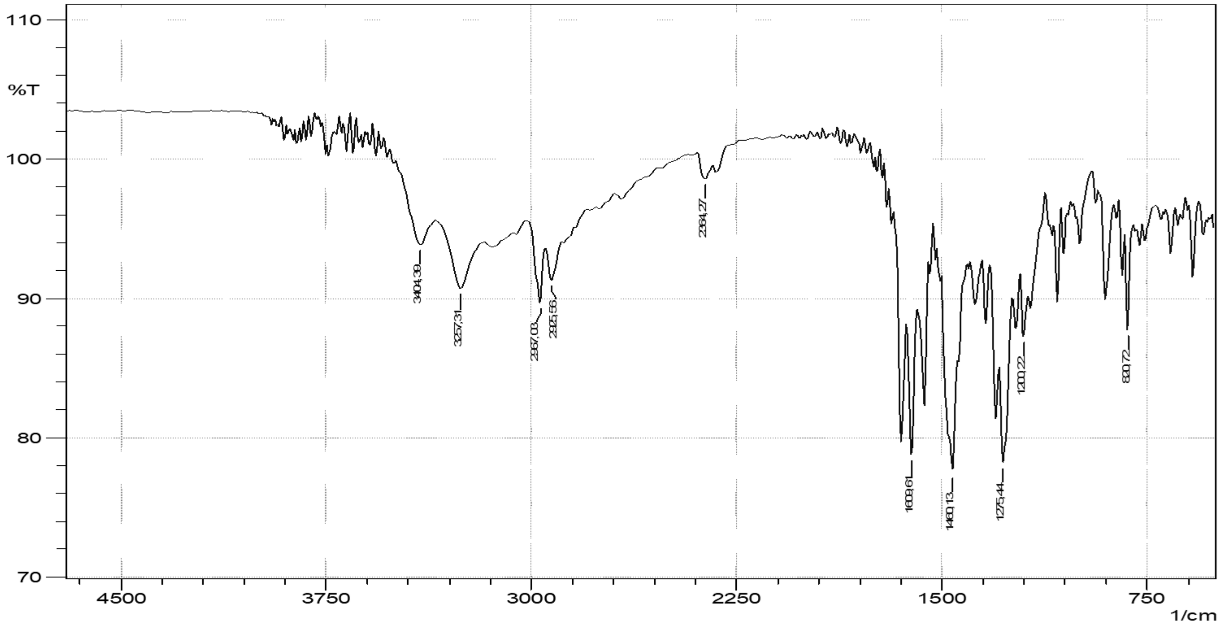

IR spectrum of GAL1 compound that isolated from *G. atroviridis* leaves

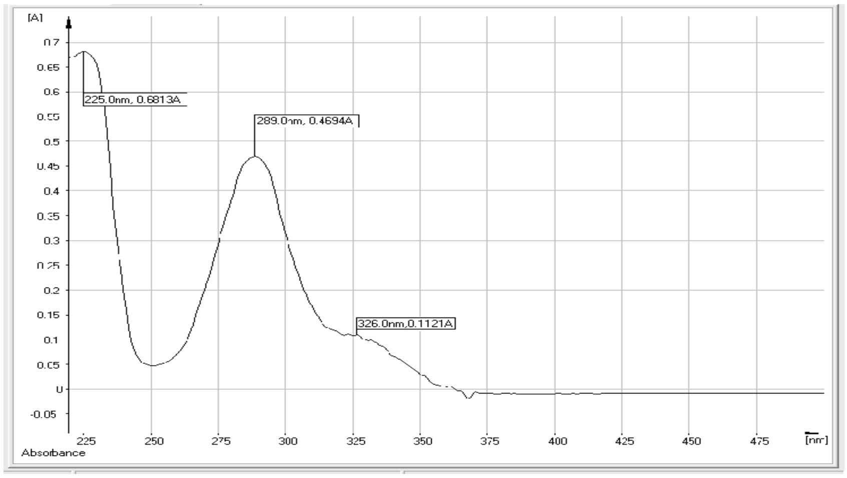

UV spectrum of GAL1 compound that isolated from *G. atroviridis* leaves

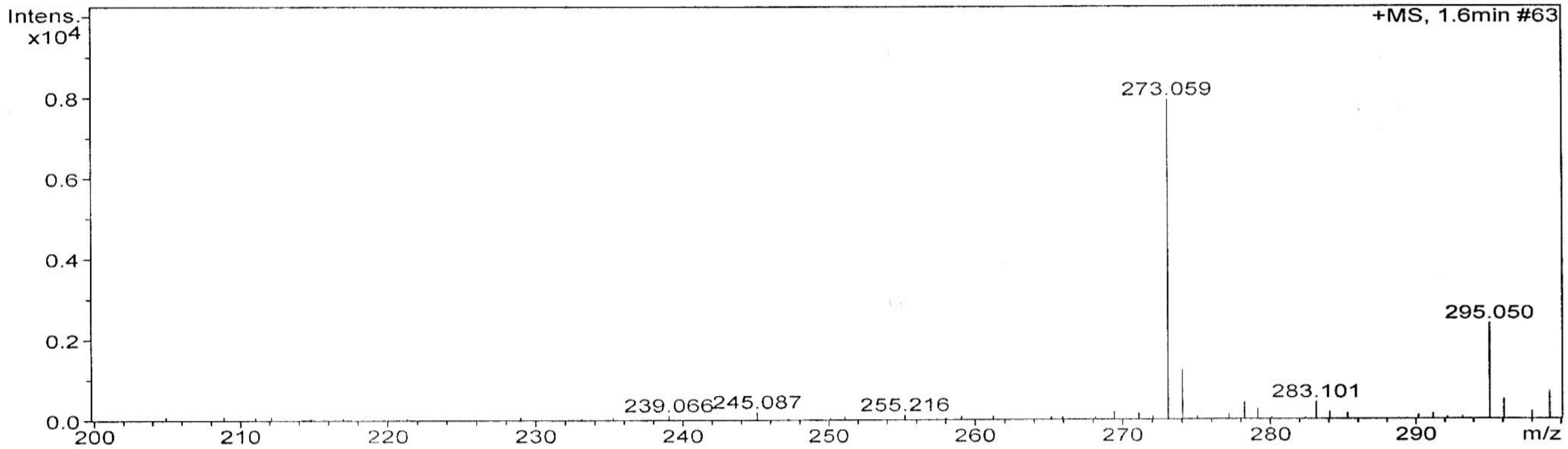

Negative Mode [M-H]+ of Mass Spectrum Using ESIMS-Trap-Direct Injection in MeOH of GAL1 compound that isolated from *G. atroviridis* leaves

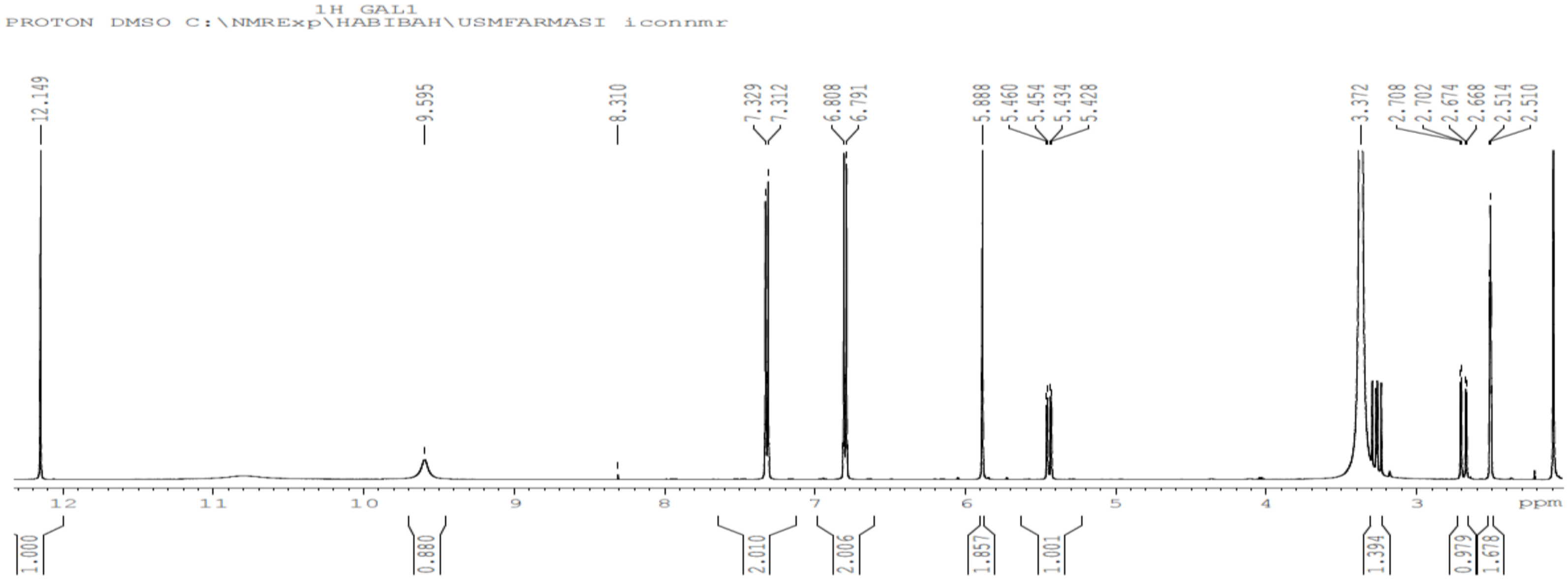

1H-NMR spectrum of GAL1 compound that isolated from *G. atroviridis leaves* using DMSO-d_6 solvent

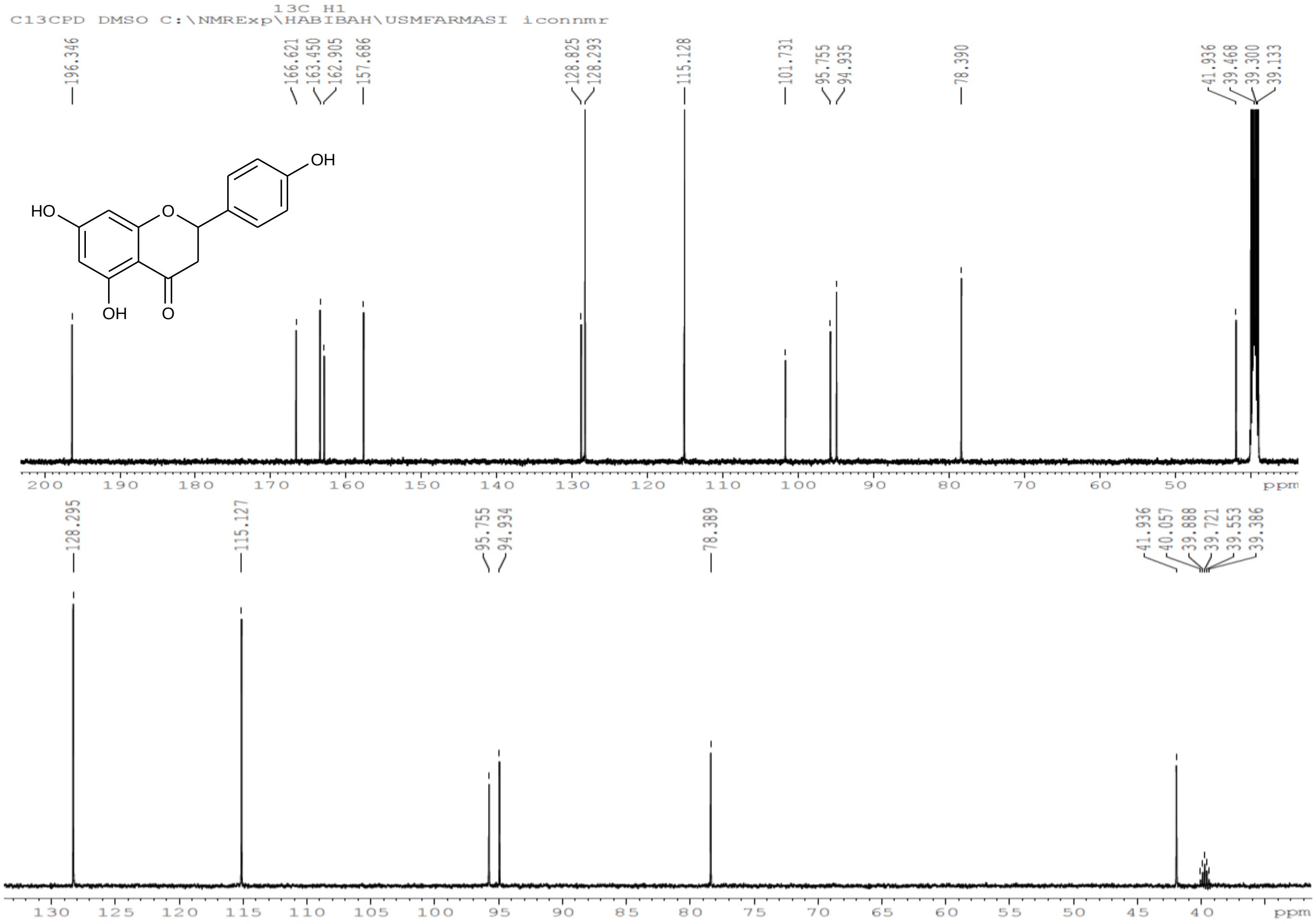

^{13}C and DEPT45-NMR (top to bottom) spectrum of GAL1 compound that isolated from *G. atroviridis leaves* using DMSO-d6 solvent

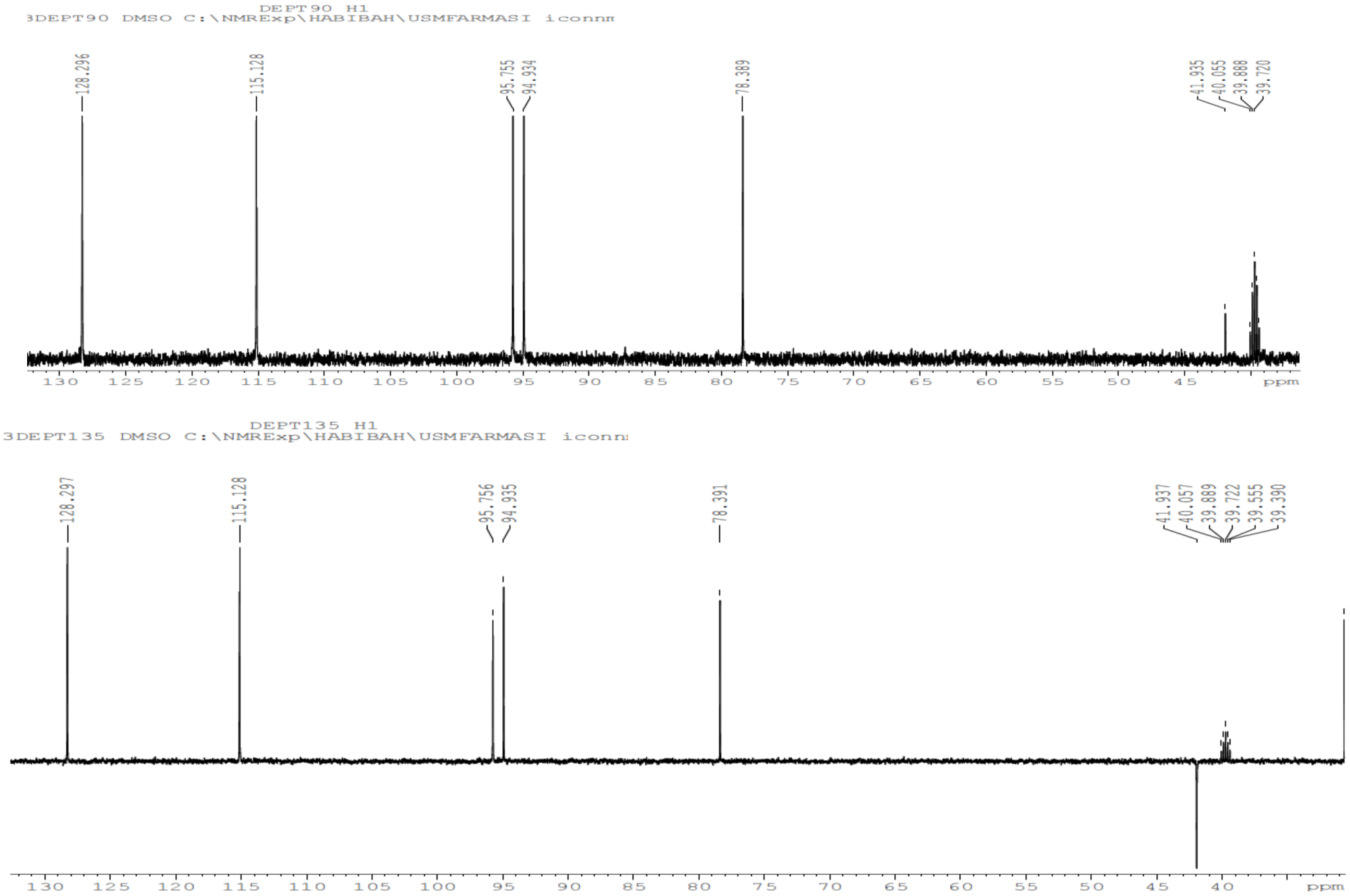

DEPT90 and DEPT135-NMR (top to bottom) spectrum of GAL1 compound that isolated from *G. atroviridis* leaves using DMSO-d6 solvent

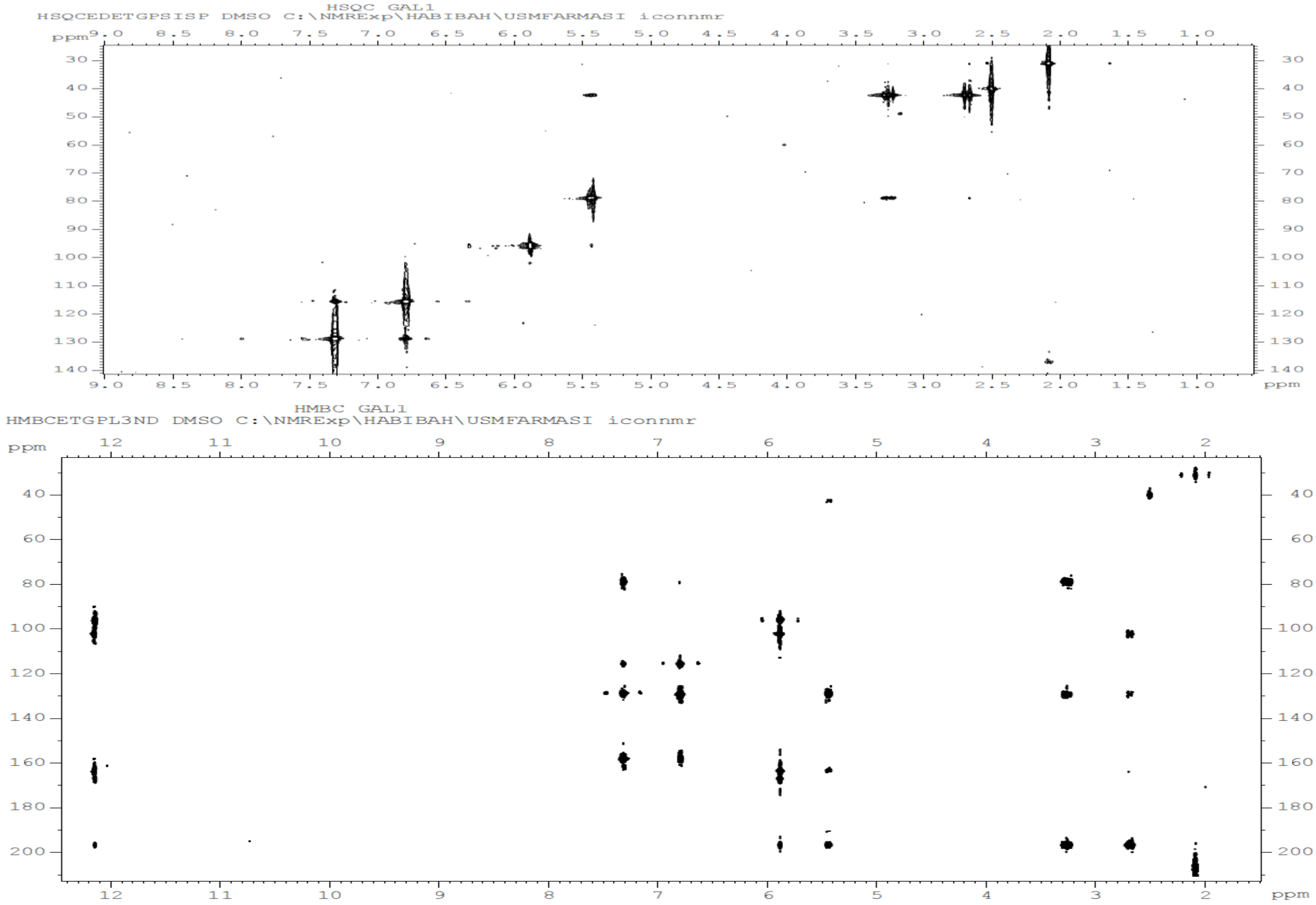

2D-HSQC and 2D-HMBC-NMR of GAL1 using DMSO-d6 solvent

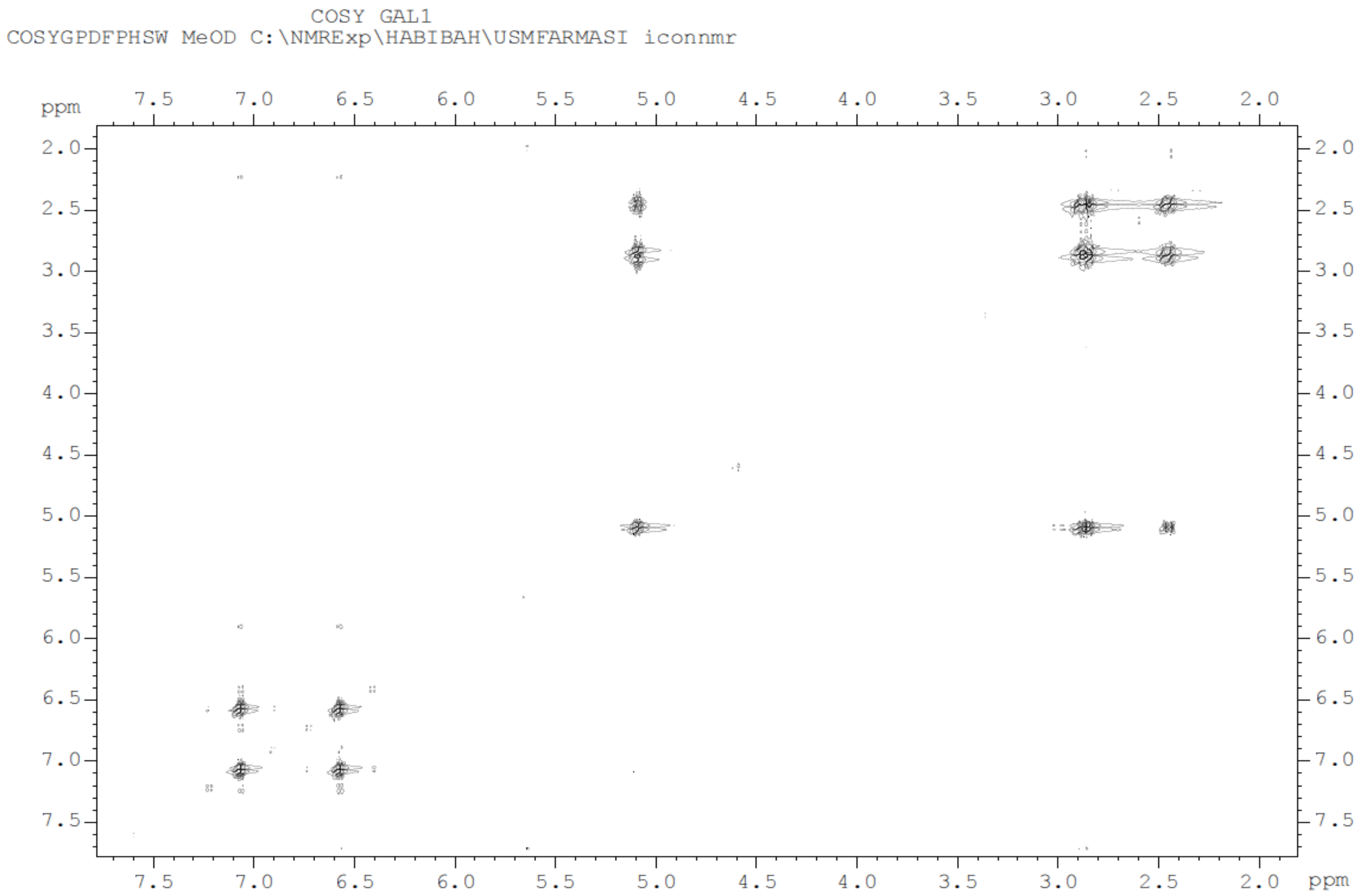

2D-COSY-NMR of GAL1 using DMSO-d6 solvent

Figure S2: Spectroscopy data of GAL1
